# Supplementary material for: Boosting immune response against cervical cancer: A combined approach using oncolytic virus and targeted therapies
Source: PLoS One. 2025 May 22;20(5):e0312979. doi: 10.1371/journal.pone.0312979 (PMC12097584; doi:10.1371/journal.pone.0312979)
Supplement: Attachment — Submitted filename: revised Response to Reviewers.docx [file pone.0312979.s008.docx]

**Response to Reviewers' Comments**
Manuscript ID: PONE-D-24-45377
Title: "Boosting Immune Response Against Cervical Cancer: A Combined Approach Using Oncolytic Virus and Targeted Therapies"

Dear Dr. Burger and the Reviewers,

We appreciate your time and insightful comments on our manuscript. We have thoughtfully reviewed all of your feedback and recommendations. Below, we present a comprehensive reply to each of the reviewers' comments and summarize the relevant changes made to the manuscript.

**Reviewer #1**

**Comment 1: Mechanistic insights into autophagy and mTOR pathways**

The reviewer suggested that we include assays, such as Western blotting for autophagy and mTOR pathway markers, to better understand how the combination therapy affects these pathways.

**Response:**
We appreciate the reviewer’s suggestion. Unfortunately, due to constraints in resources and time, it was not feasible to perform Western blotting or other mechanistic assays during this study. However, our current results strongly indicate the involvement of autophagy and mTOR pathways based on the observed synergistic effects of NDV, Everolimus, and Beclin-1. These findings align with existing literature, as cited in the Discussion, which outlines their individual and combined mechanisms in modulating these pathways.

**Comment 2: Efficacy of monotherapies**
The reviewer noted that the manuscript lacks a comprehensive analysis of the individual therapies (NDV, Everolimus, Beclin-1) and their respective efficacies.

**Response:**
We concur with the reviewer that a thorough comparison of monotherapy and combination therapy is crucial. In reply, we have broadened the Results section to incorporate a detailed comparison of tumor growth and survival among all treatment groups, showcasing the comparative effectiveness of NDV, Everolimus, and Beclin-1 as individual agents (highlighted section). Our findings indicate that although NDV alone decreased tumor volume by 35%, Everolimus and Beclin-1 resulted in reductions of 28% and 25%, respectively. The triple therapy, nevertheless, led to a 70% decrease in tumor volume. These comparisons underscore the synergistic effects of the combination therapy.

**Comment 3: Tumor volume measurement accuracy**
The reviewer recommended using in vivo imaging techniques, such as bioluminescence or fluorescence imaging, for more precise tumor volume measurements.

**Response:**
While we acknowledge the value of in vivo imaging techniques such as bioluminescence, our facility does not have access to this technology. We have relied on established caliper measurements, validated against prior studies in similar models, for tumor volume assessment. To ensure accuracy, all measurements were performed in a blinded manner and verified independently by two researchers.

**Comment 4: Histological analysis**
The reviewer suggested including immunohistochemical (IHC) analysis of tumor samples to assess immune cell infiltration, apoptosis, and proliferation.

**Response:**
We recognize the importance of IHC analysis in elucidating the immune and tumor microenvironment. However, limitations in resources and time precluded us from performing these assays. Instead, we provided indirect evidence of immune activation through cytokine analyses and CD8+ T cell proliferation assays. These findings, supported by relevant literature (e.g., Dorostkar et al., 2021), strongly suggest enhanced immune cell infiltration and activation in the combination therapy group

**Comment 5: Toxicity and safety evaluation**
The reviewer requested that we provide a detailed toxicity analysis, including body weight monitoring and liver enzyme levels.

**Response:**
In the Results section (tumor volume section 258-262), we have provided a detailed toxicity evaluation, which encompasses body weight tracking, liver enzyme measurements (ALT and AST), along with hematological assessments. Our findings indicate that there are no notable negative impacts on body weight or liver function, and blood parameters stayed within normal limits, confirming that the triple therapy is safe and well-tolerated in the mouse model.

**Comment 6: Immune dynamics over time**
The reviewer suggested measuring cytokines and immune cell infiltration at multiple time points to capture the dynamics of immune activation.

**Response:**
While we agree that longitudinal immune profiling would enhance the study, logistical constraints limited our ability to perform time-course analyses. Instead, we assessed cytokine levels and immune responses at a single, clinically relevant time point, seven days post-treatment. This provides a snapshot of immune activation and is consistent with the endpoints used in similar studies.

**Response to Minor Comments from Reviewer #1**

**Comment 1: The introduction mentions potential mechanisms but lacks specific references, especially for the roles of NDV and Beclin-1 in cancer.**

**Response:**
We have revised the Introduction to include specific references highlighting the roles of NDV and Beclin-1 in cancer therapy. For instance, NDV’s oncolytic and immune-modulatory effects (Huang et al., 2022) and Beclin-1’s role in enhancing autophagy and tumor sensitivity (Pérez-Hernández et al., 2019) are now discussed in detail.

**Comment 2: The figure legends should more clearly explain the significance markers (e.g., *P < 0.05, **P < 0.01) to make it easier for readers to interpret the data. Additionally, the legends could specify which groups are being compared for each statistical marker.**

**Response:**
I appreciate this important recommendation. We have updated the figure legends to provide clear interpretations of the significance markers (*P < 0.05, **P < 0.01) and identified the treatment groups being compared in every figure. For instance, in Figure 1A (Tumor Growth Curves), it is noted that comparisons occur between the NDV + Everolimus + Beclin-1 group and various treatment groups, with the associated significance levels marked. Comparable alterations have been implemented for all figures to guarantee clarity in understanding statistical comparisons.

**Comment 3: Some figures (e.g., cytokine levels) show mean ± SD, while others do not specify error bars. Ensure that error bars are consistent and explicitly state in figure captions whether they represent standard deviation or standard error.**

**Response:**
We have examined all data and confirmed that the error bars remain uniform across the manuscript. In the figure legends, we now clearly indicate whether the error bars signify standard deviation (SD). we clarify mean ± SD were applied to all cytokine measurements, and similar notes have been provided for the other figures too.

**Comment 4: The abstract and introduction sections are dense with technical terms (e.g., "tumor-infiltrating CD8+ T cells," "immunogenic cell death"). Simplifying some language or briefly defining key terms would improve readability for a broader audience, particularly for interdisciplinary readers.**

**Response:**
We have made the language in the Abstract and Introduction sections clearer by offering concise definitions of essential technical terms. For instance, we have included a concise description of “immunogenic cell death (ICD)” and “tumor-infiltrating CD8+ T cells” to simplify these ideas for a wider audience. Moreover, we revised certain sentences to enhance clarity and readability while maintaining scientific integrity. (highlighted part at introductions)

**Comment 5: The methods section clarifies the titering method and specific titers used for NDV to ensure reproducibility. Adding information on how NDV was quantified (e.g., PFU/mL or EID50) would help other researchers replicate the experiment.**

**Response:**
We have explained the techniques for NDV quantification in the Materials and Methods part. In particular, we included details on how NDV was tittered through the 50% embryo infectious dose (EID50) method, and we specified the NDV titer concentration utilized for treatment (10⁸ PFU/mL) in the experiment. This enhancement guarantees reproducibility and offers explicit protocols for coming researchers. (Virus Preparation section in material and methods 109-110)

**Comment 6: The ethics statement in the main text should consistently mention ethical approval details, including the institution’s name, approval code, and any specific animal welfare guidelines followed.**

**Response:**
We have revised the Ethics Statement in the Materials and Methods section to incorporate the complete details of the ethical approval. In particular, we included the institution's name (Guilan University) and the approval code (IR.GUMS.REC.1399.540). We have also cited the particular animal welfare regulations adhered to during the experiment, aligning with both institutional and national standards.

**Comment 7: The manuscript does not address the potential impact of anesthesia on immune function. Some anesthetics, such as ketamine, can modulate immune responses. Briefly discussing or controlling for any possible confounding effects would strengthen the study's conclusions.**

**Response:**
I appreciate you bringing this to my attention. We have added a brief note in the Materials and Methods section discussing the potential impact of anesthesia (ketamine) on immune function. The short duration of anesthesia during tumor implantation and sampling is unlikely to have influenced the immune outcomes.

**Comment 8: Add a concise explanation in the introduction of why these three agents were chosen for combination. For example, describe how NDV's oncolytic effect complements the immune-modulatory effects of Everolimus and Beclin-1 in targeting cervical cancer.**

**Response:**
We have updated the Introduction to give a brief rationale for choosing NDV, Everolimus, and Beclin-1 for the combination. In particular, we illustrate how the oncolytic characteristics of NDV encourage immune activation and the destruction of tumor cells, whereas Everolimus decreases tumor cell survival by focusing on the mTOR pathway, and Beclin-1 promotes autophagic cell death, positioning it as a potent adjuvant to amplify the oncolytic and immune-modulatory impacts of NDV and Everolimus. We think this explanation aids in understanding the reasoning for the combined therapy (66-82).

**Comment 9: Some abbreviations (e.g., DAMPs, TAA) are not defined upon first mention, which may need to be clarified for readers unfamiliar with these terms. Ensure all abbreviations are fully defined the first time they appear.**

**Response:**
We have revised the Introduction and Materials and Methods sections to make sure that all abbreviations are explained at their first occurrence. For example, DAMPs (damage-associated molecular patterns) and TAAs (tumor-associated antigens) are clearly defined upon their initial mention in the text, enhancing the manuscript's accessibility for readers who may not be familiar with these terms.

**Comment 10: The discussion briefly mentions the potential for clinical studies but could expand on specific challenges for clinical translation, such as optimizing dosing regimens or addressing potential toxicity in humans. This would give a balanced view of the translational feasibility of this combination therapy.**

**Response:**

**We appreciate this insightful suggestion and have expanded the Discussion to address potential challenges for clinical translation. Specifically, we highlight the need for optimizing dosing regimens to balance efficacy and safety, as well as addressing potential toxicity in humans. These challenges, while significant, are not insurmountable and can be tackled through phased clinical trials. For example, dose-escalation studies and pharmacokinetic analyses will help refine the therapeutic window of this combination therapy.**

**Reviewer #2**

**Comment 1: Discussion of similar studies**
Reviewer #2 requested that we compare our results with studies such as Dorostkar et al. (2021), Kazeruni et al. (2024), and Lee et al. (2023), focusing on cytokine levels, tumor growth inhibition, and immune responses.

**Response:**
We have updated the Discussion section and also highlighte them to feature a thorough comparison of our results with the referenced studies. We have examined the parallels and distinctions between our method and those employed in Dorostkar et al., Kazeruni et al., and Lee et al. (refer to Discussion section). Specifically, we assessed cytokine levels (IFN-γ, IL-4) and tumor growth suppression alongside the findings from these studies. Our findings align with theirs, especially about the improvement of immune responses through combination therapies, and we highlight the innovative approach of combining NDV with mTOR inhibition and autophagy modification.

**Comment 2: Clear definition of treatment groups**
The reviewer requested a clearer description of the treatment groups and what each group was treated with.

**Response:**
We have specified the treatment groups in the Materials and Methods section, offering a more comprehensive description of the treatment protocols and their administration

**Conclusion**

We have thoroughly attended to all the reviewer feedback and made the required changes to enhance the manuscript's clarity and depth. We are confident that the modifications improve the scientific integrity and clarity of our research, and we hope the updated manuscript is now appropriate for publication.

Thank you once more for your considerate and useful feedback.

Best regards,
